# Epidemiology of Onychomycosis in the United States Characterized Using Molecular Methods, 2015–2024

**DOI:** 10.3390/jof10090633

**Published:** 2024-09-05

**Authors:** Aditya K. Gupta, Tong Wang, Shruthi Polla Ravi, Avantika Mann, Sara A. Lincoln, Hui-Chen Foreman, Wayne L. Bakotic

**Affiliations:** 1 Division of Dermatology, Department of Medicine, Temerty Faculty of Medicine, University of Toronto, Toronto, ON M5S 3H2, Canada; 2 Mediprobe Research Inc., London, ON N5X 2P1, Canada; twang@mediproberesearch.com (T.W.); spollaravi@mediproberesearch.com (S.P.R.); amann@mediproberesearch.com (A.M.); 3 Bako Diagnostics, Alpharetta, GA 30005, USA; slincoln@bakodx.com (S.A.L.); hforeman@bakodx.com (H.-C.F.); wbakotic@bakodx.com (W.L.B.)

**Keywords:** onychomycosis, tinea unguium, dermatophytosis, mycology, molecular diagnosis, histopathology

## Abstract

Onychomycosis is a recalcitrant fungal infection of the nail unit that can lead to secondary infections and foot complications. Accurate pathogen identification by confirmatory testing is recommended to improve treatment outcomes. In this study, we reviewed the records of 710,541 patients whose nail specimens were sent to a single molecular diagnostic laboratory between 2015 and 2024. PCR testing revealed a more comprehensive spectrum of pathogens than previously reported, which was corroborated by the demonstration of fungal invasion on histopathology. Consistent with our current understanding, the *T. rubrum* complex (54.3%) are among the most common pathogens; however, a significant portion of mycology-confirmed diagnoses were caused by the *T. mentagrophytes* complex (6.5%), *Aspergillus* (7.0%) and *Fusarium* (4.5%). Females were significantly more likely to be infected with non-dermatophytes molds (NDMs; OR: 2.0), including *Aspergillus* (OR: 3.3) and *Fusarium* (OR: 2.0), and yeasts (OR: 1.5), including *Candida albicans* (OR: 2.0) and *C. parapsilosis* (OR 1.6), than males. The *T. mentagrophytes* complex became more prevalent with age, and conversely the *T. rubrum* complex became less prevalent with age. Patients aged ≥65 years also demonstrated a higher likelihood of contracting onychomycosis caused by NDMs (OR: 1.6), including *Aspergillus* (OR: 2.2), *Acremonium* (OR: 3.5), *Scopulariopsis* (OR: 2.9), *Neoscytalidium* (OR: 3.8), and yeasts (OR: 1.8), including *C. albicans* (OR: 1.9) and *C. parapsilosis* (OR: 1.7), than young adults. NDMs (e.g., *Aspergillus* and *Fusarium*) and yeasts were, overall, more likely to cause superficial onychomycosis and less likely to cause dystrophic onychomycosis than dermatophytes. With regards to subungual onychomycosis, *Aspergillus*, *Scopulariopsis* and *Neoscytalidium* had a similar likelihood as dermatophytes. The advent of molecular diagnostics enabling a timely and accurate pathogen identification can better inform healthcare providers of appropriate treatment selections and develop evidence-based recommendations.

## 1. Introduction

Toenail onychomycosis represents a difficult-to-treat form of superficial fungal infections [1]. The management of this chronic, slow-progressive condition is complicated by long treatment durations (often more than a year) and a high risk of recurrence (20–25%) [2]. The prevalence of onychomycosis is estimated at 4% globally, with a disproportionate burden among special populations [3]. With a myriad of treatment options available, from oral and topical antifungals to over-the-counter products and lasers, complete cure rates for onychomycosis (i.e., eradication of fungal infection with normal-appearing nails) remain sub-optimal, highlighting the need for tailored treatments [2,4].

Onychomycosis is primarily caused by dermatophytes with *Trichophyton rubrum* being the most frequently reported pathogen. However, an infection by non-dermatophyte molds (NDMs) and yeasts are considered negative prognostic factors owing to their varied antifungal susceptibility profiles (e.g., terbinafine vs. itraconazole as first-line treatments), as well as diagnostic challenges that can cause treatment delays [5,6,7,8]. Traditional onychomycosis diagnosis by fungal culture has significant limitations due to the slow turnaround time (2–4 weeks) and low sensitivity; with NDMs and yeasts, there is an added challenge of requiring repeated culture isolations, which can further delay treatment initiation [9,10]. 

In recent years, molecular diagnostics by polymerase chain reaction (PCR) has become available for the speciation of infecting fungal agents. A multiplexed PCR assay design, in particular, can simultaneously detect multiple fungal agents with a 1–2-day turnaround time. The combined use of PCR with histopathologic examination has been recommended as a reliable diagnostic method as it enables pathogen identification with a direct demonstration of nail plate invasion [11]. In this study, we aim to update our current understanding of the epidemiology of onychomycosis in the United States by using a combination of molecular diagnosis through a multiplex real-time PCR assay and histopathologic examination.

## 2. Materials and Methods

Records of all nail specimens submitted to a CLIA-certified, molecular diagnostic laboratory (Bako Diagnostics, Alpharetta, GA, USA) were reviewed. This cohort represents clinically suspected onychomycosis patients seen by dermatologists and podiatrists across the United States. The inclusion criteria were specimens subjected to confirmatory testing by multiplex real-time PCR and histopathologic examination as per a physician’s order; specimens subjected to a single test only, or by fungal culture, were excluded. This study constitutes a review of secondary, de-identified diagnostic data. All procedures were provided by a CLIA-certified laboratory as a part of routine care; as such, these do not represent a clinical trial for which ethics oversight and informed consent are required. 

### 2.1. Multiplex Real-Time PCR

DNA extraction and multiplex real-time PCR (BakoDx Onychodystrophy Agent Detection) were performed, as previously described [12,13]. DNA extracts were subjected to two sequential multiplex real-time PCR panels for detection and identification (QuantStudio^TM^ 6, Life Technologies, Carlsbad, CA, USA). The detection panel included dermatophytes, NDMs, and yeasts. The dermatophyte identification panel included the *T. rubrum* complex (*T. rubrum*, *T. violaceum*), the *T. mentagrophytes* complex (*T. benhamiae*, *T. indotineae*, *T. interdigitale*, *T. mentagrophytes*, *T. tonsurans*), *Microsporum* and *Epidermophyton*. The NDM identification panel included *Acremonium*, *Alternaria*, *Aspergillus*, *Curvularia*, *Fusarium*, *Scopulariopsis* and *Neoscytalidium*. The yeast identification panel included *Candida albicans*, *C. guilliermondii*, *C. parapsilosis*, *C. tropicalis*, *Cryptococcus*, *Malassezia*, and *Trichosporon*.

### 2.2. Histopathology

**Figure 1:**
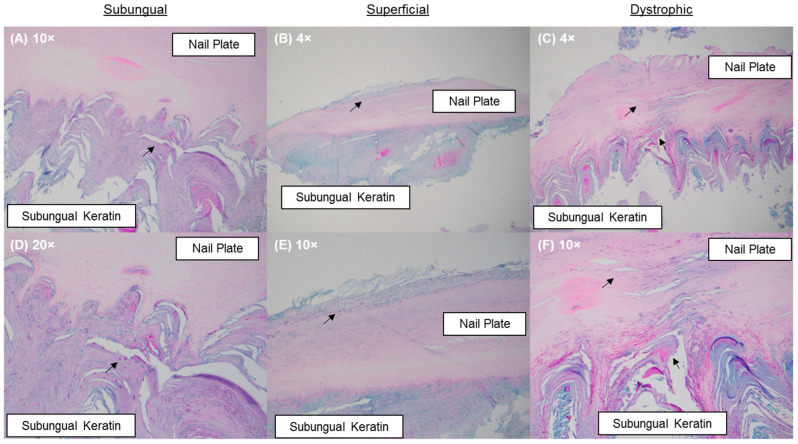
PAS-stained nail specimens: (**A**,**D**) a subungual infection pattern evidenced by the presence of fungal elements in the subungual keratin; (**B**,**E**) a superficial infection pattern evidenced by the presence of fungal elements within the dorsal aspect of the nail plate; and (**C**,**F**) a dystrophic infection pattern evidenced by the presence of fungal elements in the nail plate and in the subungual keratin. Fungal elements are indicated by black arrows.

Nail specimens were stained by Periodic acid–Schiff (PAS) and/or Grocott’s methenamine silver (GMS) and reviewed by a dermatopathologist. Infection patterns were identified as either subungual (infiltrating fungal elements within the subungual keratin only); superficial (infiltrating fungal elements across the superficial keratin layers of the nail plate); or dystrophic (infiltrating fungal elements through the nail plate and subungual keratin) (Figure 1). Fungal element quantities were categorized as rare (sparse fungal elements primary in the subungual region with little to no association with nail keratin); minimal (<10% of submitted nail keratin involvement); moderate (10–80% of submitted nail keratin involvement); or florid (>80% of submitted nail keratin involvement) [12].

### 2.3. Data Analysis

A positive, mycology-confirmed onychomycosis diagnosis was defined as samples testing positive by PCR with demonstration of fungal invasion on histopathologic examination. The analysis of patient characteristics was restricted to unique patients; repeat samples were removed. For patients with multiple specimens collected, only the first specimen was included. Analysis of histopathologic examination results included all specimens. Mixed infections were not analyzed due to the inability to match positive histopathologic results with one or the other fungal agents detected. 

Data curation and analyses were performed using Microsoft Excel (version 2301). Patient demographics (age, sex, U.S. census region), PCR, and histopathologic examination results were tabulated. Relationships between patient characteristics, pathogen identification results and histopathologic examination results were quantified using the odds ratio (OR) and 95% confidence interval (CI); two-sided *p*-values were calculated as previously described by Altman and Bland [14]. A *p*-value of 0.05 was considered statistically significant. 

## 3. Results

**Figure 2:**
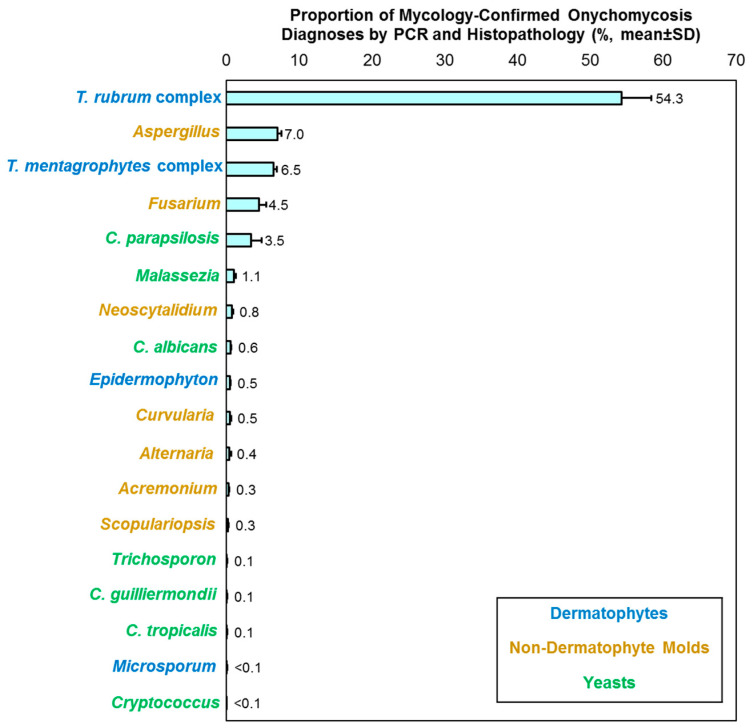
Distribution of fungal agents in patients with mycology-confirmed onychomycosis diagnoses by PCR and histopathology. Results are stratified by year and presented as mean ± SD; mixed detections and un-speciated samples are not shown.

Records were retrieved from 710,541 patients (797,560 specimens), with a clinical diagnosis of onychomycosis, from March 2015 to April 2024 (9 years and 2 months). When assessing results stratified by year, the mean positivity rate—per PCR and histopathologic examination—was 51.0% (standard deviation [SD]: 2.8). Dermatophytes consistently accounted for the highest proportion of mycology-confirmed onychomycosis diagnoses (Mean [SD]: 63.7% [4.6]; Range, 55.4–69.0%) followed by NDMs (Mean [SD]: 20.1% [4.9]; Range, 14.6–30.7%), and yeasts (Mean [SD]: 6.2% [0.8]; Range, 4.5–6.8%). The *T. rubrum* complex were the most common dermatophytes detected, *Aspergillus* and *Fusarium* were the most common NDMs detected, and *C. parapsilosis* was the common yeast detected (Figure 2). No significant temporal changes were observed.

### 3.1. Sex Differences

As shown in Table 1, Table 2 and Table 3, females exhibited a 70% lower odds of contracting dermatophyte onychomycosis than males (OR: 0.3 [95% CI: 0.3, 0.3], *p* < 0.001). Conversely, females were twice as likely to contract NDM onychomycosis (OR: 2.0 [95% CI: 1.9, 2.0], *p* < 0.001), and had 50% higher odds of contracting yeast onychomycosis (OR: 1.5 [95% CI: 1.5, 1.6], *p* < 0.001), than males.

Among NDMs, *Aspergillus* and *Fusarium* were the most common. Females were three times more likely to be detected with *Aspergillus* (OR: 3.3 [95% CI: 3.1, 3.4], *p* < 0.001), and twice as likely to be detected with *Fusarium* (OR: 2.0 [95% CI: 1.9, 2.1], *p* < 0.001), than males. Similarly, for yeasts, females were twice as likely to be detected with *C. albicans* (OR: 2.0 [95% CI: 1.8, 2.2], *p* < 0.001), as well as 60% higher odds for *C. parapsilosis* (OR: 1.6 [95% CI: 1.6, 1.7], *p* < 0.001), than males. 

### 3.2. Age Differences

An age-dependent increase was observed concerning onychomycosis caused by *T. mentagrophytes* complex, NDMs, and yeasts (Table 1, Table 2 and Table 3). Among dermatophytes, the elderly (≥65 years) showed 50% lower odds (OR: 0.5 [95% CI: 0.5, 0.5], *p* < 0.001) of contracting onychomycosis caused by the *T. rubrum* complex than young adults (18–44 years). Conversely, this patient group showed an almost 4-fold increase in the likelihood of contracting onychomycosis caused by the *T. mentagrophytes* complex (OR: 3.9 [95% CI: 3.7, 4.1], *p* < 0.001). Similar findings were seen for older adults (45–64 years) compared to younger adults (18–44 years). The *T. mentagrophytes* complex was significantly less likely to be found among children (OR: 0.2 [95% CI: 0.2, 0.3], *p* < 0.001).

An age-dependent increase was observed for NDM onychomycosis. The elderly were more likely to contract onychomycosis caused by *Aspergillus* (OR: 2.2 [95% CI: 2.1, 2.3], *p* < 0.001), *Acremonium* (OR: 3.5 [95% CI: 2.8, 4.4], *p* < 0.001), *Scopulariopsis* (OR: 2.9 [95% CI: 2.3, 3.6], *p* < 0.001), and *Neoscytalidium* (OR: 3.8 [3.2, 4.4], *p* < 0.001), but not *Fusarium* (OR: 0.7 [95% CI: 0.7, 0.7], *p* < 0.001), compared to young adults. For yeasts, both *C. albicans* (OR: 1.9 [95% CI: 1.7, 2.2], *p* < 0.001) and *C. parapsilosis* (OR: 1.7 [1.6, 1.8], *p* < 0.001) were more frequently detected in the elderly compared to young adults. 

### 3.3. Regional Differences

No significant regional differences were found overall for dermatophytes, NDMs, yeasts, and mixed infections (Table 1, Table 2 and Table 3). Among the NDMs, *Aspergillus* was more commonly detected in the U.S. South compared to the Midwest (OR: 1.7 [95% CI: 1.6, 1.8], *p* < 0.001). In contrast, *Acremonium* (OR: 0.8 [95% CI: 0.7, 0.9], *p* = 0.005) and *Scopulariopsis* (OR: 0.5 [95% CI: 0.5, 0.6], *p* < 0.001) were less likely to be detected in the U.S. South. *Neoscytalidium* was more likely to be detected in the U.S. Northeast (OR: 4.8 [95% CI: 3.9, 5.9], *p* < 0.001), South (OR: 3.0 [2.4, 3.7], *p* < 0.001) and West (OR: 1.8 [95% CI: 1.4, 2.4], *p* < 0.001), compared to the Midwest. 

Among yeasts, no regional differences were found for *C. albicans*. For *C. parapsilosis*, it was more likely to be detected in the U.S. Northeast (OR: 1.5 [95%: 1.4, 1.6], *p* < 0.001) and South (OR: 1.5 [95%: 1.4, 1.6], *p* < 0.001), compared to the Midwest.

### 3.4. Differentiation of NDMs and Yeasts on Histopathologic Examination

**Figure 3:**
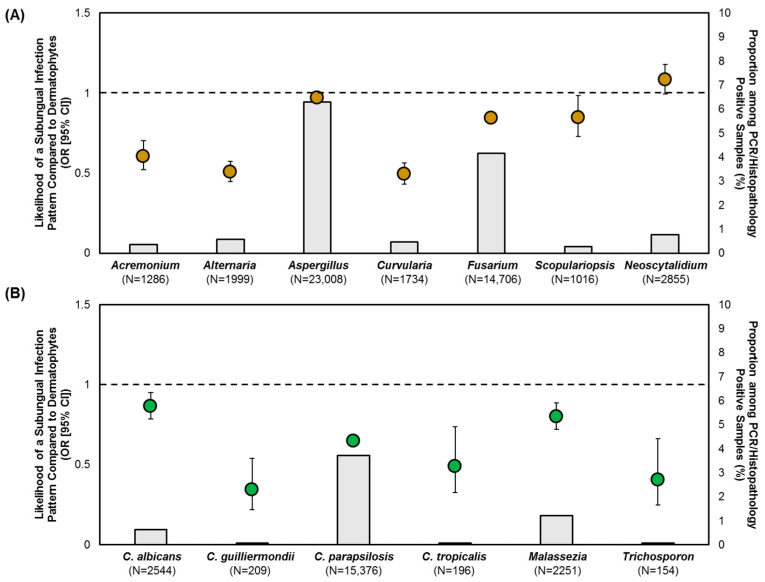
Likelihood of: (**A**) NDMs; and (**B**) yeasts in causing subungual onychomycosis, with moderate-to-florid fungal element quantities, compared to dermatophytes. Data points represent OR (95% CI), grey-shaded bars represent the prevalence of the respective NDM/yeast target, the reference (dotted) line represents dermatophytes with an OR of 1.

**Figure 4:**
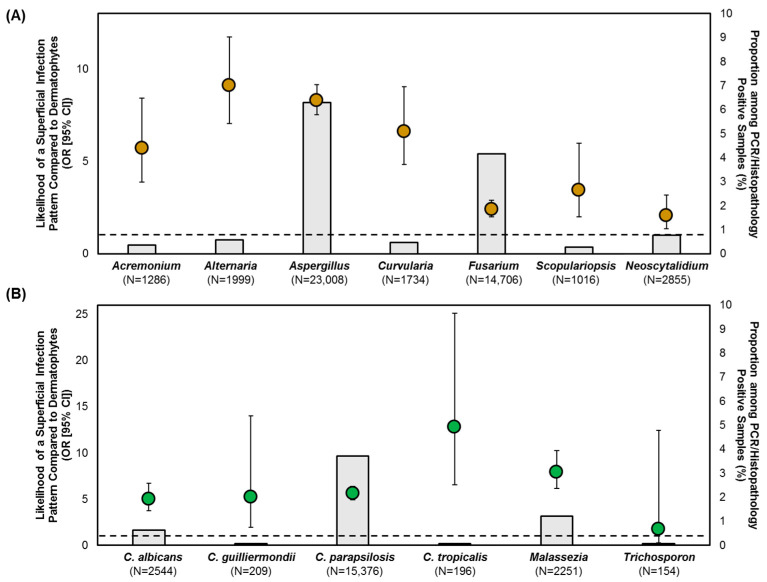
Likelihood of: (**A**) NDMs; and (**B**) yeasts in causing superficial onychomycosis compared to dermatophytes. Data points represent OR (95% CI), grey-shaded bars represent the prevalence of the respective NDM/yeast target, the reference (dotted) line represents dermatophytes with an OR of 1.

**Figure 5:**
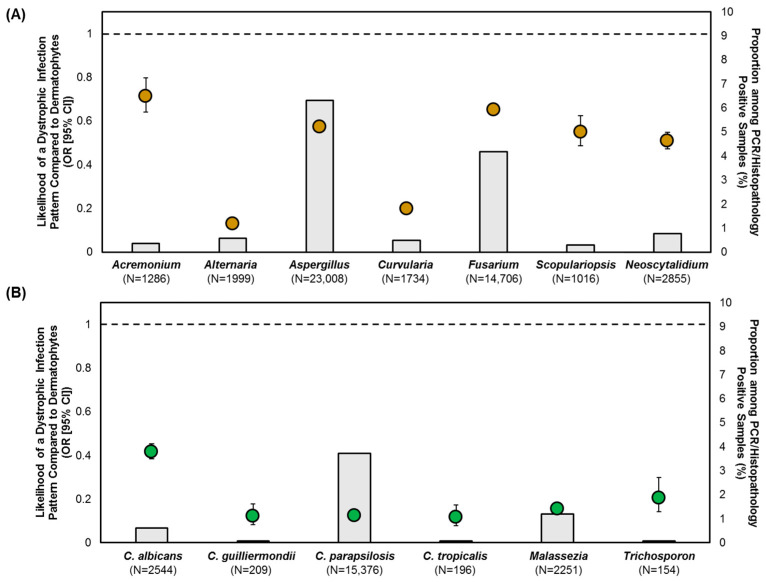
Likelihood of: (**A**) NDMs; and (**B**) yeasts in causing dystrophic onychomycosis compared to dermatophytes. Data points represent OR (95% CI), grey-shaded bars represent the prevalence of the respective NDM/yeast target, the reference (dotted) line represents dermatophytes with an OR of 1.

To assess the pathogenic potentials of NDMs and yeasts, the likelihoods of causing subungual, superficial, or dystrophic onychomycosis were quantified with dermatophytes as the reference group (Figure 3, Figure 4 and Figure 5). Due to the distinct possibility of contaminant or commensal organisms in the subungual keratin (Figure 1), a positive NDM or yeast specimen with the subungual infection pattern was only considered if moderate-to-florid fungal element quantities were present. All samples exhibiting a superficial or dystrophic patterned infection were included in the analysis, as fungal elements were observed to penetrate the nail plate (Figure 1).

After controlling for moderate-to-florid fungal element quantities, *Aspergillus*, *Scopulariopsis* and *Neoscytalidium* exhibited a similar degree of likelihood (*p* > 0.05) in causing a subungual patterned infection as dermatophytes (Figure 3A). *Fusarium* exhibited a 16% lower likelihood than dermatophytes (OR: 0.84 [95% CI: 0.81, 0.88], *p* < 0.001). In contrast, all yeasts were less likely to cause a subungual infection (Figure 3B). The highest relative probability was found in *C. albicans* (OR: 0.86 [95% CI: 0.79, 0.95], *p* = 0.002).

All NDMs and yeasts were more likely to cause a superficial patterned infection, and less likely to cause a dystrophic patterned infection, than dermatophytes (Figure 4 and Figure 5). However, we observed differences in likelihood between NDM and yeast species. *Fusarium*, *Scopulariopsis* and *Neoscytalidium* demonstrated a lower likelihood of causing a superficial infection (OR: 2.1–3.5) compared to *Acremonium*, *Aspergillus*, *Alternaria*, and *Curvularia* (OR: 5.7–9.1) (Figure 4A). *Alternaria* and *Curvularia* had a lower likelihood of causing a dystrophic infection (OR: 0.13–0.2) than all other NDM species (*Acremonium*, *Aspergillus*, *Fusarium*, *Scopulariopsis*, *Neoscytalidium*) (OR: 0.51–0.71) (Figure 5A). *C. albicans* (OR: 0.42) could be differentiated from non-*albicans Candida* (OR: 0.12) as well as *Malassezia* (OR: 0.16) and *Trichosporon* (OR: 0.21), with a higher likelihood of causing dystrophic onychomycosis (Figure 5B). 

## 4. Discussion

Treatment decision-making for onychomycosis patients often does not take pathogen identification into consideration [15,16]; this is partially due to a lack of uptake in confirmatory testing and methodological limitations [17,18]. Although not commonly found in onychomycosis patients, an infection by NDMs or yeasts is more difficult to treat [5]. In view of the current treatment paradigm often necessitating long-term maintenance and prophylactic regimens—with varying monthly costs ($8–$1542), efficacy rates and safety concerns—an accurate identification of the causative pathogen should be considered to optimize treatment outcome and improve patient satisfaction [2,4,19]. 

In this study, we report a detailed analysis of the pathogen spectrum in onychomycosis patients by PCR diagnosis, which was corroborated based on the demonstration of fungal invasion on histopathologic examination. Historically, the rise in onychomycosis is closely linked to the intercontinental spread of *T. rubrum* replacing *T. mentagrophytes* as the dominant pathogen in the 20th century [20]. However, our results showed that the *T. rubrum* complex was only detected in 54.3% of the mycology-confirmed cases. Mono-infections caused by *Aspergillus* accounted for 7.0% of the mycology-confirmed cases, followed by *Fusarium* at 4.5%, and *C. parapsilosis* at 3.5% (Figure 2). A similar relative distribution was observed in previous epidemiology studies conducted in the United States and Canada [21,22].

Due to differences in methodology—especially if there is a lack of repeat sampling to confirm the presence of NDMs or yeasts isolated on culture, or the lack of a histopathologic examination to demonstrate hyphal invasion—our findings are not directly comparable to other epidemiological surveys of onychomycosis. However, a survey in France (2018–2019) found a rise in cases of *Fusarium* toenail onychomycosis (13.2% [16/121]) [23], which were detected by fungal culture and identified by MALDI-TOF mass spectrometry; a survey in Spain (2020–2021) found a high prevalence of *Candida* spp. in onychomycosis involving the hallux (13.2% [5/38]) [24], which was detected by fungal culture and identified by PCR.

Furthermore, we identified trends suggesting that these underrecognized pathogens are more likely to be detected in specific patient groups and geographical regions. Even though male patients have been traditionally considered to have a higher risk for contracting onychomycosis [20], there was a differential pathogen profile between the sexes. Specifically, males were significantly more likely to have a dermatophyte infection, whereas females were more likely to contract NDM onychomycosis by *Aspergillus* and *Fusarium* as well as *Candida* onychomycosis (Table 1, Table 2 and Table 3). *Aspergillus* was more frequently found in the U.S. South. Through comparison of infection patterns on histopathology, *Aspergillus* was just as likely to cause a subungual infection as dermatophytes, followed by *Fusarium* (Figure 3A). All NDMs were more likely to cause superficial onychomycosis than dermatophytes. Both *Aspergillus* and *Fusarium* were less likely to cause a dystrophic infection than dermatophytes (Figure 5); however, their likelihood was higher compared to the other NDMs detected. 

The underlying cause for the higher likelihood of NDM and yeast onychomycosis in female patients remains unclear. It can be postulated from previous case studies that this may be due to a higher likelihood of nail (micro)trauma secondary to the use of ill-fitting, open-toed footwear [25,26,27]. Hirose et al. reviewed 26 cases of *Aspergillus* onychomycosis reported across Japan, of which 25 cases were female patients [25]. A recent review also identified a higher propensity for females to contract *Fusarium* onychomycosis than males (66.3% vs. 24.4%), but the reasons are not specified [6].

Although advanced age has been consistently reported as a significant risk factor for developing onychomycosis [3], we found an age-dependent decrease in the prevalence of the *T. rubrum* complex and inversely an age-dependent increase in the *T. mentagrophytes* complex, NDMs, and yeasts (Table 1, Table 2 and Table 3). Among NDMs, we found a higher propensity for *Aspergillus*, *Acremonium*, *Scopulariopsis* and *Neoscytalidium* infections in the 45–64-year and ≥65-year age groups. For yeasts, *C. albicans* and *C. parapsilosis* were also more prevalent in the 45–64-year and ≥65-year age groups. Poor peripheral circulation, comorbidities, brittle nails, and increased (micro)trauma are predisposing factors in this patient group. Further research is warranted to explain these differential detection rates.

*Candida* onychomycosis is thought to affect predominately fingernails causing opportunistic infections [28]. Its diagnostic criteria is ambiguous [10], and its pathogenic potential is unclear. In this study, we found yeasts (e.g., *C. albicans*, non-*albicans Candida*) to more likely cause superficial onychomycosis, and to less likely cause subungual or dystrophic onychomycosis, than dermatophytes. *C. albicans*, in particular, stands out with the highest relative likelihood of causing dystrophic onychomycosis (Figure 5B). This finding is in agreement with the study by Otašević et al. [29], in which 137 cases of *Candida* finger- and toenail onychomycosis were analyzed; *C. albicans* was the only species found in patients presenting with nail dystrophy and paronychia, which was attributed to its potential of transforming into hyphal forms. Other surveillance studies have reported similar findings, where *Candida* onychomycosis is more common among females and the elderly, with *C. parapsilosis* being the dominant *Candida* species [30,31,32]. 

Taken together, the results of the present study revealed that a significant portion of mycology-confirmed onychomycosis diagnoses are caused by underrecognized pathogens that disproportionately affect females and the elderly. Contrary to our current understanding of onychomycosis, females—who are underrepresented in clinical trials—should not be considered as a low-risk group owing to their higher propensity for contracting NDM and yeast infections [33]. Although older patients are generally at a higher risk for onychomycosis, they were less likely to be affected by the dominant dermatophytic pathogen (*T. rubrum* complex). The goal of onychomycosis treatment should be to eradicate the fungal infection [11]; a rapid diagnosis by PCR-enabling pathogen identification, with infection pattern characterization by histopathology, provides healthcare providers with the opportunity to make tailored treatment decisions.

### Limitations

Our findings are limited by the retrospective study design and diagnostic samples that were submitted to a single laboratory. Given that confirmatory testing is currently offered to only 15.3% of suspected onychomycosis patients [18], our cohort may overrepresent patients with a higher disease burden than the background population. Other predisposing factors to developing onychomycosis, such as comorbidities, family history, occupations and social determinants [34,35,36], could not be analyzed in the present study; further works are much needed to update our current understanding of the epidemiology of onychomycosis. We also recognize that PCR testing for onychomycosis is currently more widely available in developed countries; continued advocacy efforts are warranted to expand the access of this platform in place of fungal culture to improve diagnostic effectiveness.

Lastly, in order to decrease the risk of detecting NDMs or yeasts as non-pathogenic organisms in the subungual space without association to the nail plate or nail keratin invasion, we attempted to correlate PCR results with histopathologic findings both in terms of infection pattern (Figure 1) and fungal element quantities. Specifically, when a subungual patterned infection is observed concurrent with the detection of NDMs or yeasts, we restricted the dataset to only specimens with moderate-to-florid fungal element quantities; in other words, specimens with rare-to-minimal fungal element quantities were excluded if they showed a subungual patterned infection, as we judged these samples to have more potential to represent commensal organisms or contaminants. However, the same restriction was not applied to samples exhibiting a superficial or dystrophic patterned infection since they clearly demonstrate the presence of fungal elements within the nail plate keratin, indicative of keratinolytic activities (Figure 1). In cases where rare or minimal fungal growth is in association with nail keratin invasion and histopathology-defined disease, we judged this observation to be more likely a result of the sampling artifact (i.e., the submitted nail specimen represents an infection area with a reduced fungal load than the rest of the affected nail plate).

When the analysis included all subungual patterned infections, irrespective of fungal element quantities, or only in samples with rare-to-minimal quantities, we observed that most, if not all, the NDMs exhibited a higher likelihood of causing a subungual infection than dermatophytes, which was likely due to a higher prevalence of non-pathogens (Gupta et al., unpublished data [37]). In contrast, when we applied the moderate-to-florid fungal element quantities restriction (Figure 3), *Aspergillus*, *Fusarium*, *Scopulariopsis* and *Neoscytalidium* exhibited higher likelihoods—indicating a higher pathogenic potential—than *Acremonium*, *Alternaria* and *Curvularia*. This approach remains to be clinically validated with treatment outcomes.

## 5. Conclusions

Onychomycosis is a significant global healthcare burden complicated by an ever-changing pathogen spectrum that underlies treatment challenges. Although NDMs and yeasts are traditionally considered as uncommon pathogens, we identified a higher risk skewed towards female patients and the elderly. To our knowledge, clinical trials have not been conducted to assess the treatment efficacy of dermatophyte onychomycosis vis-à-vis NDM or yeast onychomycosis; the advent of rapid molecular diagnostics by PCR, with infection pattern characterization by histopathology, may assist healthcare providers in developing evidence-based recommendations.

## Figures and Tables

**Table 1:** Likelihood of contracting **dermatophyte** onychomycosis per patient sex, age group, and U.S. census region.

Variable	Dermatophytes			*T. rubrum* Complex			*T. mentagrophytes* Complex		
	N	%	OR (95% CI)	N	%	OR (95% CI)	N	%	OR (95% CI)
**Sex**									
Male	128,751	46.7	Referent	111,612	40.5	Referent	10,985	4.0	Referent
Female	98,889	23.0	**0.3 (0.3, 0.3)**	82,095	19.1	**0.3 (0.3, 0.4)**	12,283	2.9	0.7 (0.7, 0.7)
**Age group**									
<18 years	5477	27.7	0.6 (0.6, 0.6)	5210	26.4	0.6 (0.6, 0.7)	55	0.3	**0.2 (0.2, 0.3)**
18–44 years	57,888	39.2	Referent	53,446	36.2	Referent	2029	1.4	Referent
45–64 years	83,877	32.2	0.7 (0.7, 0.7)	72,695	27.9	0.7 (0.7, 0.7)	7144	2.7	**2.0 (1.9, 2.1)**
≥65 years	81,948	29.5	0.7 (0.6, 0.7)	63,577	22.9	**0.5 (0.5, 0.5)**	14,239	5.1	**3.9 (3.7, 4.1)**
**Region**									
Northeast	72,646	34.7	1.1 (1.1, 1.1)	62,818	30.0	1.1 (1.1, 1.2)	6617	3.2	0.9 (0.9, 0.9)
Midwest	34,005	32.2	Referent	28,827	27.3	Referent	3676	3.5	Referent
South	103,046	30.4	0.9 (0.9, 0.9)	86,997	25.7	0.9 (0.9, 0.9)	10,836	3.2	0.9 (0.9, 1.0)
West	19,791	34.9	1.1 (1.1, 1.2)	16,617	29.3	1.1 (1.1, 1.1)	2334	4.1	1.2 (1.1, 1.3)

**OR ≤ 0.5 or ≥1.5 are shown in bold.**

**Table 2:** Likelihood of contracting **non-dermatophyte mold** onychomycosis per patient sex, age group and U.S. census region.

Variable	NDMs			*Aspergillus*			*Fusarium*		
	N	%	OR (95% CI)	N	%	OR (95% CI)	N	%	OR (95% CI)
**Sex**									
Male	19520	7.1	Referent	3206	1.5	Referent	2933	1.4	Referent
Female	55556	12.9	**2.0 (1.9, 2.0)**	15,621	4.7	**3.3 (3.1, 3.4)**	9057	2.7	**2.0 (1.9, 2.1)**
**Age group**									
<18 years	1226	6.2	0.7 (0.7, 0.8)	53	0.3	**0.1 (0.1, 0.2)**	309	2.0	0.8 (0.7, 0.9)
18–44 years	12,198	8.3	Referent	2666	2.3	Referent	3019	2.6	Referent
45–64 years	27,993	10.7	1.3 (1.3, 1.4)	6185	3.1	1.4 (1.3, 1.4)	4906	2.5	1.0 (0.9, 1.0)
≥65 years	34,407	12.4	**1.6 (1.5, 1.6)**	10,152	4.8	**2.2 (2.1, 2.3)**	3859	1.8	0.7 (0.7, 0.7)
**Region**									
Northeast	17,127	8.2	0.8 (0.7, 0.8)	3876	2.4	0.9 (0.9, 1.0)	3436	2.1	0.8 (0.8, 0.9)
Midwest	10,976	10.4	Referent	2118	2.6	Referent	2086	2.6	Referent
South	42,599	12.6	1.2 (1.2, 1.3)	11,317	4.4	**1.7 (1.6, 1.8)**	6121	2.4	0.9 (0.9, 1.0)
West	4926	8.7	0.8 (0.8, 0.9)	1651	3.7	1.4 (1.3, 1.5)	464	1.0	**0.4 (0.4, 0.4)**
**Variable**	***Acremonium***			***Scopulariopsis***			***Neoscytalidium***		
	**N**	**%**	**OR (95% CI)**	**N**	**%**	**OR (95% CI)**	**N**	**%**	**OR (95% CI)**
**Sex**									
Male	449	0.2	Referent	339	0.2	Referent	939	0.4	Referent
Female	541	0.2	0.8 (0.7, 0.9)	445	0.1	0.8 (0.7, 1.0)	1232	0.4	0.8 (0.8, 0.9)
**Age group**									
<18 years	3	0.0	**0.3 (0.1, 0.8)**	8	0.1	0.7 (0.3, 1.5)	4	0.0	**0.2 (0.1, 0.5)**
18–44 years	87	0.1	Referent	84	0.1	Referent	178	0.2	Referent
45–64 years	363	0.2	**2.5 (1.9, 3.1)**	274	0.1	**1.9 (1.5, 2.5)**	825	0.4	**2.7 (2.3, 3.2)**
≥65 years	542	0.3	**3.5 (2.8, 4.4)**	430	0.2	**2.9 (2.3, 3.6)**	1194	0.6	**3.8 (3.2, 4.4)**
**Region**									
Northeast	243	0.1	0.7 (0.5, 0.8)	184	0.1	**0.5 (0.4, 0.6)**	996	0.6	**4.8 (3.9, 5.9)**
Midwest	184	0.2	Referent	195	0.2	Referent	104	0.1	Referent
South	457	0.2	0.8 (0.7, 0.9)	335	0.1	**0.5 (0.5, 0.6)**	983	0.4	**3.0 (2.4, 3.7)**
West	111	0.2	1.1 (0.9, 1.4)	79	0.2	0.7 (0.6, 1.0)	103	0.2	**1.8 (1.4, 2.4)**

**OR ≤ 0.5 or ≥1.5 are shown in bold. NDM, non-dermatophyte mold.**

**Table 3:** Likelihood of contracting **yeast** onychomycosis per patient sex, age group and U.S. census region.

Variable	Yeasts			*C. albicans*			*C. parapsilosis*		
	N	%	OR (95% CI)	N	%	OR (95% CI)	N	%	OR (95% CI)
**Sex**									
Male	6806	2.5	Referent	549	0.2	Referent	3849	1.4	Referent
Female	15,928	3.7	**1.5 (1.5, 1.6)**	1719	0.4	**2.0 (1.8, 2.2)**	9698	2.3	**1.6 (1.6, 1.7)**
**Age group**									
<18 years	373	1.9	0.9 (0.8, 1.0)	22	0.1	**0.5 (0.3, 0.8)**	186	0.9	0.7 (0.6, 0.8)
18–44 years	3238	2.2	Referent	313	0.2	Referent	2007	1.4	Referent
45–64 years	8631	3.3	**1.5 (1.5, 1.6)**	843	0.3	**1.5 (1.3, 1.7)**	5286	2.0	**1.5 (1.4, 1.6)**
≥65 years	10,832	3.9	**1.8 (1.7, 1.9)**	1113	0.4	**1.9 (1.7, 2.2)**	6251	2.3	**1.7 (1.6, 1.8)**
**Region**									
Northeast	6866	3.3	1.3 (1.2, 1.3)	660	0.3	1.0 (0.9, 1.1)	4480	2.1	**1.5 (1.4, 1.6)**
Midwest	2754	2.6	Referent	342	0.3	Referent	1497	1.4	Referent
South	11,902	3.5	1.4 (1.3, 1.4)	1109	0.3	1.0 (0.9, 1.1)	6998	2.1	**1.5 (1.4, 1.6)**
West	1398	2.5	0.9 (0.9, 1.0)	166	0.3	0.9 (0.8, 1.1)	693	1.2	0.9 (0.8, 0.9)

**OR ≤ 0.5 or ≥1.5 are shown in bold.**

## Data Availability

Restrictions apply to the availability of these data. Data were obtained from Bako Diagnostics (Alpharetta, GA, USA) and are available from A.K.G. with the permission of Bako Diagnostics.
